# Validation of the PHQ-9 as a screening instrument for depression in diabetes patients in specialized outpatient clinics

**DOI:** 10.1186/1472-6963-10-235

**Published:** 2010-08-12

**Authors:** Kirsten M van Steenbergen-Weijenburg, Lars de Vroege, Robert R Ploeger, Jan W Brals, Martijn G Vloedbeld, Thiemo F Veneman, Leona Hakkaart-van Roijen, Frans FH Rutten, Aartjan TF Beekman, Christina M van der Feltz-Cornelis

**Affiliations:** 1 Department of Diagnostics and Treatment of Common Mental Disorders, Trimbos-Institute, PO Box 725, 3500 AS Utrecht, the Netherlands; 2 Department of Psychology and Psychiatry, ZGT Almelo, PO Box 7600, 7600 SZ Almelo, the Netherlands; 3 Department of Internal Medicine, ZGT Hengelo, PO Box 546, 7550 AM Hengelo, the Netherlands; 4 Institute for Medical Technology Assessment. Erasmus MC, University Medical Centre Rotterdam Burgemeester Oudlaan 50, PO Box 1738, 3000 DR Rotterdam, The Netherlands; 5 Department of Psychiatry, VU University Medical Centre, De Boelelaan 1117, PO Box 7057, 1007 MB Amsterdam, The Netherlands; 6 Deptartment of Developmental, Clinical and Cross-cultural Psychology, University of Tilburg, PO Box 90153, 5000 LE Tilburg, the Netherlands

## Abstract

**Background:**

For the treatment of depression in diabetes patients, it is important that depression is recognized at an early stage. A screening method for depression is the patient health questionnaire (PHQ-9). The aim of this study is to validate the 9-item Patient Health Questionnaire (PHQ-9) as a screening instrument for depression in diabetes patients in outpatient clinics.

**Methods:**

197 diabetes patients from outpatient clinics in the Netherlands filled in the PHQ-9. Within 2 weeks they were approached for an interview with the Mini Neuropsychiatric Interview. DSM-IV diagnoses of Major Depressive Disorder (MDD) were the criterion for which the sensitivity, specificity, positive- and negative predictive values and Receiver Operator Curves (ROC) for the PHQ-9 were calculated.

**Results:**

The cut-off point of a summed score of 12 on the PHQ-9 resulted in a sensitivity of 75.7% and a specificity of 80.0%. Predictive values for negative and positive test results were respectively 93.4% and 46.7%. The ROC showed an area under the curve of 0.77.

**Conclusions:**

The PHQ-9 proved to be an efficient and well-received screening instrument for MDD in this sample of diabetes patients in a specialized outpatient clinic. The higher cut-off point of 12 that was needed and somewhat lower sensitivity than had been reported elsewhere may be due to the fact that the patients from a specialized diabetes clinic have more severe pathology and more complications, which could be recognized by the PHQ-9 as depression symptoms, while instead being diabetes symptoms.

## Background

Seven percent of adults in the USA have been diagnosed with Major Depressive Disorder (MDD) and in adults with chronic diseases, such as diabetes, this increases to over eleven percent [1]. Although the causal connection between the two remains unclear, the consequences are far-reaching. Having both diabetes and depression is associated with poor glycaemic control, resulting in more severe complications and a lower quality of life [2,3]. With the increasing severity of diabetes, the prevalence of depression also increases, and especially in vulnerable patients such as those with diabetes-related complications. Depression has severe consequences; underlining the importance of focus on the prevention of depression.

Depression often remains unrecognized, and although several screening questionnaires are available, unfortunately, most questionnaires have been validated for use in primary care in patients with less complex medical illnesses. It is expected that patients with severe diabetes and depression are frequently present in specialized outpatient clinics or hospitals, but specialists often do not have the necessary time or skills to recognize depression. The recognition of depression is very important and the Patient Health Questionnaire (PHQ-9) was developed for this purpose [4,5]. This instrument has already been validated for primary care patients, cardiac patients in general hospitals [6], and diabetes patients in primary care [7], but not for diabetes patients in specialized outpatient clinics. Besides that, the present study is the first one assessing operating characteristics for the PHQ-9 in diabetes patients. In patients with chronic medical diseases, co-morbid MDD can be difficult to identify, because the symptoms of the two may overlap. The effect of symptom overlap on the performance of screening instruments for depression, such as the PHQ-9 [4], would be that higher cut-off points are necessary to correctly identify MDD in the chronically ill than in a population with less severe illnesses. The overall effect would be that both sensitivity and specificity would decline.

In this study we assessed the criterion validity, in terms of sensitivity, specificity, positive and negative predictive value. What is new is that Receiver Operator Curves (ROC) were assessed of the PHQ-9 for MDD in diabetes patients in specialized outpatient clinics. These specialized clinics differ from general diabetic care clinics in that in these specialized clinics foremost patients with severe diabetes with complications are present and specialized clinical diabetes care is provided by a team of a diabetologist, a specialized diabetes nurse and a dietician.

## Methods

### Patients and procedures

After approval of the study protocol by the Medical Ethics Committee "Verenigde commissies mensgebonden onderzoek", patients were selected from two specialized outpatient clinics for diabetes in the east of the Netherlands.

After giving informed consent, the patients received the PHQ-9 by mail. Those who gave informed consent had a MIni Neuropsychiatric Interview (MINI) by telephone [8], within two weeks after filling in the PHQ-9. The interviews were administered by trained interviewers who were not blinded for the PHQ-9 scores. Patients were excluded if we were unable to contact them within two weeks after they had filled in the PHQ-9 or if they did not give informed consent.

### Measurements

The PHQ-9 is a screening questionnaire, developed by Kroenke et al [4] containing nine questions about the symptoms of MDD. It has the following answer categories: "not at all", "various days", "more than half the days" and "almost every day". Respectively zero, one, two or three points were scored and a summed score of the nine questions was calculated. The questions refer to the situation in the previous two weeks. This questionnaire is based on the Diagnostic and Statistic Manual of mental disorders-IV (DSM-IV) criteria for diagnosing MDD in patients with medical illnesses, and the questions concerning fatigue, concentration, depressive complaints, thoughts of death, etc. The PHQ-9 can also be used to screen patients for MDD specifically, according to the DSMIV criteria. This 'algorithm', developed by Kroenke et al [4] is positive for MDD when a total of five questions on the PHQ-9 have a score of two or more points, with exception for question nine: scoring at least 1 point is sufficient. Besides that, question one ("in the past two weeks I had less interest and fun in doing activities"), or question two ("in the past two weeks I felt dejected, depressed or desperate"), have to be answered positively.

In this study, the telephone based MINI [8] was used as the criterion instrument to diagnose MDD. The questions in this interview, which are often used in clinical practice, are based on the DSMIV criteria.

### Data-analyses

The criterion validity of the PHQ-9 was analyzed in terms of sensitivity, specificity, and positive and negative predictive values for different cut-off scores. As shown in Table 1, sensitivity is the proportion of those with MDD, according to the MINI who are correctly screened out by the PHQ (a/a+c). Specificity is the proportion of those without MDD, according to the MINI, who are correctly identified as such by the PHQ (d/b+d). The positive predictive value is the proportion of those with a positive (elevated) PHQ-9 score who have MDD according to the MINI (a/a+b). The negative predictive value is the proportion of those with a negative (normal) PHQ-9 score who do not have MDD (d/d+c), as shown in table 1.

**Table 1:** Sensitivity and specificity calculations

Clinical diagnosis	Test result		
	**MINI positive**	**MINI negative**	**Total**

PHQ-9 positive	A	B	A+B

PHQ-9 negative	C	D	C+D

Total	A+C	B+D	A+B+C+D

Sensitivity = A/(A+C)

Specificity = D/(D+B)

Predictive value (positive test results) = A/(A+B)

Predictive value (negative test results) = D/(D+C)

There is always a possibility that a patient is falsely screened positive or negative. Therefore, it is important to reduce this possibility by identifying the most optimal combination of sensitivity and specificity. This way, the clinically acceptable risk of a falsely screened patient can be determined.

When a higher criterion value is selected, the false positive fraction will decrease with increased specificity but on the other hand the true positive fraction and sensitivity will decrease, as described by Zweig et al [9]:

"In a Receiver Operating Characteristic (ROC) curve the true positive rate (Sensitivity) is plotted in function of the false positive rate (100-Specificity) for different cut-off points. Each point on the ROC plot represents a sensitivity/specificity pair corresponding to a particular decision threshold. A test with perfect discrimination (no overlap in the two distributions) has a ROC plot that passes through the upper left corner (100% sensitivity, 100% specificity). Therefore the closer the ROC plot is to the upper left corner, the higher the overall accuracy of the test" [9]. The flattening of the curve shows when there is no additional benefit of the screening method.

To answer the research question on the criterion validity of the PHQ-9 a Receiver Operating Characteristic curve (ROC curve) is made with SPSS version 15.0.

## Results

Of the 1,278 patients that filled in the PHQ-9, 382 were excluded because they did not give informed consent and another 501 were excluded because they did not return the PHQ-9 within 2 weeks. Of the 395 eligible patients, 198 were unable to be reached within 2 weeks after they had filled in the PHQ-9, so data on 197 participants were finally included in our analyses (49.8% of the eligible patients).

The mean age of the study sample (N = 197) was 61.82 years (SD = 13.69), 96 participants were female (48.7%) and 101 were male (51.3%). 106 patients (53.8%) scored negative on the PHQ-9 and 91 (46.2%) scored positive on the PHQ-9. The mean PHQ-9 score was 7.95 (SEM = 0.46). There were a total of 197 MINIs, 81.2% were negative for MDD (N = 160) and 18.8% were positive for MDD (N = 37). The baseline characteristics of these patients are shown in Table 2.

**Table 2:** Baseline characteristics

Age (Mean, sd)	
Years	61.82 (13.69)

**Gender (freq, %)**	

Female	96 (48.7)

Male	101 (51.3)

**PHQ-9 score (mean, std. error)**	

0-27	7.95 (0.46)

0-10 "negative"	2.78 (0.27)

>10 "positive"	14.02 (0.41)

**Algorithm score (N, %)**	

Negative	154 (78.2)

Positive	43 (21.8)

**MINI (N, %)**	

Negative	160 (81.2)

Positive	37 (18.8)

**Hospital(N, %)**	

ZGT Almelo	166 (84.3)

ZGT Hengelo	31 (15.7)

Table 3 shows the sensitivity, specificity, and predictive values for both positive and negative test results for a selected number of summed PHQ-9 scores. These were calculated for summed scores from 0 to 27, but a range of summed scores from 8 to 12 showed the most optimal results, and are therefore presented here.

**Table 3:** Sensitivity, specificity, predictive values and efficiency outcomes for different cut-off scores

	Score ≥8	Score ≥9	Score ≥10	Score ≥11	Score ≥12
	
Occurrence	N = 99 (50.3%)	N = 95 (48.2%)	N = 91 (46.2%)	N = 71 (36.0%)	N = 60 (30.5%)
Sensitivity	91.9%	91.9%	91.9%	81.1%	75.7%
Specificity	59.4%	61.9%	64.4%	74.4%	80.0%
PV (pos. t.)	96.9%	97.1%	97.2%	94.4%	93.4%
PV (neg. t.)	34.3%	35.8%	37.4%	37.4%	46.7%
Efficiency	49.8%	51.8%	53.8%	63.9%	69.5%

(Abbreviations: PV, predictive value; neg. t, negative test results; pos. t., positive test results)

The requirements for a screener can vary, but for most purposes the lower boundary of the sensitivity of a screener is around the 75%. Table 3 shows that a cut-off point of 12 combines sensitivity > 75% with the optimal specificity (80%). Lower cut-off points by definition improve the sensitivity, with a sensitivity of 91.9% for the summed scores of 8, 9 or 10 on the PHQ-9. This is at the expense of the efficiency of the screening; a lower specificity varying from 59.4% for a cut-off score of 8, to 74.4% for a cut-off score of 11. Predictive values for the test-positive results varied from 34.4% for a cut-off score of 8, to 46.7% for a cut-off score of 12. The predictive values for the test-negative results increased from 96.9% for a cut-off score of 8, to 97.2% for a cut-off score of 10, and then decreased to 93.4% for the cut-off score of 12.

Table 4 shows the results of the validation of the algorithm score of the PHQ-9. We found a sensitivity of 58.3% and a specificity of 86.9%. Predictive values for positive and negative test results were respectively 50.0% and 90.3%.

**Table 4:** Sensitivity, specificity, predictive values and efficiency outcomes for the algorithm score

	PHQ 0-27	PHQ > 10
	
Occurrence	*	N = 91
pos. algorithm	N = 42 (21.3%)	N = 42 (46.2%)
neg. algorithm	N = 155 (78.7%)	N = 49 (53.9%)

Sensitivity	58.3%	63.6%
Specificity	86.9%	63.8%
PV (pos. t.)	50.0%	50.0%
PV (neg. t.)	90.3%	75.5%
Efficiency	78.7%	53.9%

*consists of all participants, N = 197

(Abbreviations: pos. algorithm, positive algorithm; neg. algorithm, negative algorithm; PV, predictive value; neg. t, negative test results; pos. t., positive test results)

**Figure 1:**
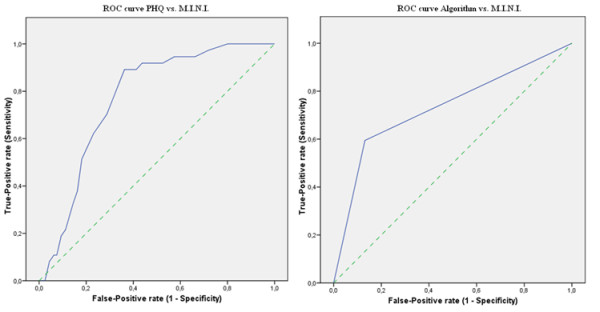
**ROC curve for PHQ-9 vs. MINI and Algorithm vs. MINI**. (Note: the dotted line is the reference line)

The ROC curves, calculated for the PHQ-9 summed score are shown in Figure 1. The calculated area under the curve (AUC) for the PHQ-9 summed score versus MINI was 0.77 (SE = 0.04; 95%CI = 0.69 - 0.84). The calculated AUC and CI scores are shown in Table 5. These values are significant.

**Table 5:** Outcomes of AUC for the PHQ-9 summed score versus the MINI

			Asymptotic 95% Confidence Interval	
Test result:	AUC	SE^a^	Lower bound	Upper bound

PHQ-9 summed score	0.77	0.04	0.69	0.84

^a ^Under the nonparametric assumption

(Abbreviations: AUC, area under curve; SE, standard error)

## Discussion

### Findings of the study

In this study, for the first time the PHQ9 is validated as a screening instrument for MDD in diabetes patients visiting a specialized outpatient clinic. As such, it gives us important information about the validity and appropriate cut off scores for identifying Diabetes patients with a high possibility for having MDD.

The main finding of this study is that the PHQ-9 appears to have satisfactory criterion validity as a screening instrument for MDD in diabetes patients in specialized outpatient clinics. We recommend using a cut-off score of 12 to recognize depression in diabetes patients from specialized outpatient clinics. This is a higher cutoff score than is generally used for identification of MDD patients in the primary care setting in patients without advanced medical co-morbidity.

The predictive value in general does not only depend on the quality of the instrument, but varies with the prevalence of the disorder in the study population and with factors that may blur diagnosis and cause misclassification of those who are at risk for the diagnosis. In the present sample, the a priori likelihood of patients having MDD was relatively high (18,8%), while their often complex medical condition might tend to blur the contrast between those with and without depression. The consequence is that one would expect screening instruments for depression to perform less efficiently, and that higher cut-off points are necessary to efficiently eliminate those with depression. This proved to be the case.

The algorithm score, although it literally follows the DSMIV criteria, did not show very good sensitivity or specificity (resp. 63.8% and 63.6%). This is unexpected, because the MINI interview, used in this study to diagnose MDD, also follows the DSMIV criteria.

### Limitations of the study

Limitations of this study were first of all that it was not possible to blind the interviewers with regard to the PHQ scores. Although they were not aware of the purpose of the interview, knowing the scores might have influenced the outcomes, and might also have inflated both sensitivity and specificity. Secondly, the response rate was not high, because only patients who returned the PHQ-9 within two weeks after receiving it were included. Unfortunately, no epidemiological data on the non responders could be obtained, as this was confidential information which the hospitals were not allowed to provide. Therefore, the findings of this study cannot be extrapolated to the general population as no indication can be made of the characteristics of the non-responders. However, this study was not intended to give information for the use of the screener in the broad population, but for the validity of the pHQ9 as a screener in patients with Diabetes visiting specialized diabetes clinics. For this purpose, the findings are very relevant.

Also, the telephone response of almost 50% might seem low, but this is an average response rate on epidemiologic research in the Netherlands.

Thirdly, there could be up to a 2-week lag between administration of the PHQ-9 and the MINI. During this time, higher PHQ-9 scores might have "regressed to the mean" thus meaning higher cut-off points might have been needed to correspond to depressive disorder diagnoses than if the PHQ-9 and MINI had been administered more closely in time.

Our findings correspond with the results of other studies. A cut-off point of ≥10 was found to be the optimal cut-off point with high sensitivity and specificity scores (respectively 91% and 89% for MDD) in stroke patients [10]. In a study in which depression was assessed in patients with traumatic brain injury, an optimal cut-off point of ≥10 with a sensitivity of 93% and a specificity of 89% was also reported [11]. In several studies performed in the medically ill, an optimal cut-off score of 12 was recommended [12,13]. One reason why we found that a higher cut-off point was optimal for patients with diabetes may be that the symptoms of MDD and diabetes can overlap.

## Conclusions

Looking closer at our results, a cut-off point of ≥10 or ≥12 can be used, depending on the purpose of the screening. If the main purpose is screening for patients with depression in a clinical setting, a cut-off point of ≥12 can be recommended because of its higher specificity. The probability that a patient is falsely screened as depressed is then at an acceptable rate. On the other hand, a cut-off point of ≥10 is best for epidemiological research, because it ensures a larger group of participants with possible depressive disorders, probably ranging in severity. As a result, we recommend the summed score of the PHQ-9 for screening for depression in diabetes patients in specialized outpatient clinics. This is a reliable questionnaire which will subsequently result in improving the quality of the patient's life.

## Competing interests

The authors declare that they have no competing interests.

## Authors' contributions

KvS performed the statistic analyses and wrote the manuscript, LdV performed statistic analyses, RP, JB, MV and TV revised the manuscript and provided information on the patients, LHvR, FR and AB revised the manuscript and reviewed the statistical analyses and CFC supervised the analyses and reviewed the manuscript. All authors read and approved the final manuscript.

## Pre-publication history

The pre-publication history for this paper can be accessed here:

http://www.biomedcentral.com/1472-6963/10/235/prepub
